# A powerful partnership: researchers and patients working together to develop a patient-facing summary of clinical trial outcome data

**DOI:** 10.1093/jamia/ocad099

**Published:** 2023-06-21

**Authors:** Emily Ruzich, Jason Ritchie, France Ginchereau Sowell, Aliyah Mansur, Pip Griffiths, Hannah Birkett, Diane Harman, Jayne Spink, David James, Matthew Reaney

**Affiliations:** Patient Centered Solutions, IQVIA, Boston, Massachusetts, USA; Patient Centered Solutions, IQVIA, New York, New York, USA; Patient Centered Solutions, IQVIA, New York, New York, USA; Patient Centered Solutions, IQVIA, London, UK; Patient Centered Solutions, IQVIA, Paris, France; Medical and Patient Communications, IQVIA, Reading, UK; Patient Centered Solutions, IQVIA, New York, New York, USA; Prostate Cancer Research, London, UK; Prostate Cancer Research, London, UK; Patient Centered Solutions, IQVIA, Reading, UK

**Keywords:** patient-reported outcome measure, qualitative research, data visualization, health communication, prostatic neoplasms

## Abstract

**Objective:**

Availability of easy-to-understand patient-reported outcome (PRO) trial data may help individuals make more informed healthcare decisions. Easily interpretable, patient-centric PRO data summaries and visualizations are therefore needed. This three-stage study explored graphical format preferences, understanding, and interpretability of clinical trial PRO data presented to people with prostate cancer (PC).

**Materials and Methods:**

A 7-day online survey exploring people with PC’s preferences for different PRO data presentations (stage 1; *n* = 30) informed development of a draft plain-language resource sheet containing PRO data. After refining for clarity during cognitive debriefing interviews (stage 2; *n* = 18), the final resource sheet was circulated to people with PC for broader feedback (stage 3; *n* = 45).

**Results:**

Although participants expressed preferences for certain graphical formats (pie charts and bar charts), preference did not always associate with interpretability and overall message clarity. Iterative development (stages 1 and 2) led to a final resource sheet, which 91.1% of participants in stage 3 considered useful and informative, and 88.9% expressed interest in receiving similar resources in the future.

**Discussion:**

Findings demonstrate PRO data are relevant to people with PC and highlights that targeted resource sheets can support patient–clinician discussions. Appropriate graphical formatting and use of plain-language text is essential for conveying interpretable PRO data. Data visualization preferences are context dependent.

**Conclusion:**

Resource sheets summarizing clinical trial PRO data can be helpful for decision-making in PC. Researchers and patients can work together to develop clear, relevant, sensitive, and understandable resource sheets, which equally consider patient priorities as well as those of scientists.

## INTRODUCTION

The Food and Drug Administration, European Medicines Agency, Department of Health and Social Care, and multiple Health Technology Agency authorities have acknowledged the importance of patient-focused drug development and committed to incorporating patient-reported clinical trial data into their assessments. Such data provide an understanding of how patients feel and function, describing symptom burden, impact on daily activities, perceived quality of life (QoL), and treatment satisfaction, among other things only known to the patient. If collected using well-defined and reliable patient-reported outcome (PRO) questionnaires, these data can inform patients of the potential value and drawbacks of treatment options as perceived by trial participants, to help with their own healthcare decision-making.^1–5^ Many trials now include at least one PRO measure.^6^ However, common approaches to synthesizing clinical trial PRO data are not typically intended for lay audiences and thus may not be optimized to help patients directly, some of whom will have low health literacy levels.^7^^,^^8^

Most PRO data communication targets medical professionals and scientists via journals and conferences, using complex statistics and academic or medical syntax. Research suggests that, in the absence of interpretable PRO data, patients turn to the internet and social media when looking for information about how a new treatment may cause them to feel or function, and to share their own lived experiences with others.^7–9^ Unfortunately, much of this information is unregulated, with quality control challenges making inaccurate, incomplete, and misleading information common.^7^^,^^8^

As a research community, we must do better to ensure clinical trial PRO data are accessible and inclusive to all—including those with limited health literacy.^7^^,^^8^ Of course, data must be accurate and scientifically robust, but should be communicated in a manner that affords audiences with varied health literacy levels the same opportunity to use such information to facilitate patient–clinician discussions and self-manage their care.^9^^,^^10^ Both the language used in communication as well as any accompanying visual displays are critically important to ensure both health literacy and visualization literacy.^11–15^

We must acknowledge the need to take different approaches when communicating to medical professionals and patients. For example, prior research has shown that patients and clinicians prefer different amounts of statistical data in the presentation of PRO data.^16^ General guidance regarding communicating clinical trial data to patients includes the use of plain language, simplified visual formats with a single central message, and avoidance of complex graphical elements.^16–19^ These previous efforts specific to PRO data are echoed by similar initiatives undertaken outside the realm of clinical outcome assessment (COA) data to improve and standardize reporting, for instance in developing a generalizable approach for communicating results of genetic testing to patients.^20^ However, there is a need for additional research to inform guidance on how best to present PRO data to specific patient populations in line with their preferences to help facilitate understanding and support patients’ engagement in their own care.^21^

In an effort to further advance guidance, people with prostate cancer (PC) supported development of a resource sheet summarizing clinical trial PRO data for patients use, providing input on its interpretability, relevance, clarity, and usability. PC is the most common cancer in the UK, and the second most common cancer diagnosis in men worldwide.^22^^,^^23^ Clinical care is routinely informed by patient priorities and preferences, and thus empowering these patients with comprehensive, relevant, meaningful, and understandable PRO data is important.^24^ The resource sheet was codeveloped by IQVIA Patient Centered Solutions (a scientific research organization) and Prostate Cancer Research (PCR; a UK-based patient advocacy organization).

The overall goal of this study was to develop a brief, informative, and understandable resource sheet summarizing PRO data from a PC clinical trial in line with presentation preferences of patients.

## MATERIALS AND METHODS

**Figure 1.**
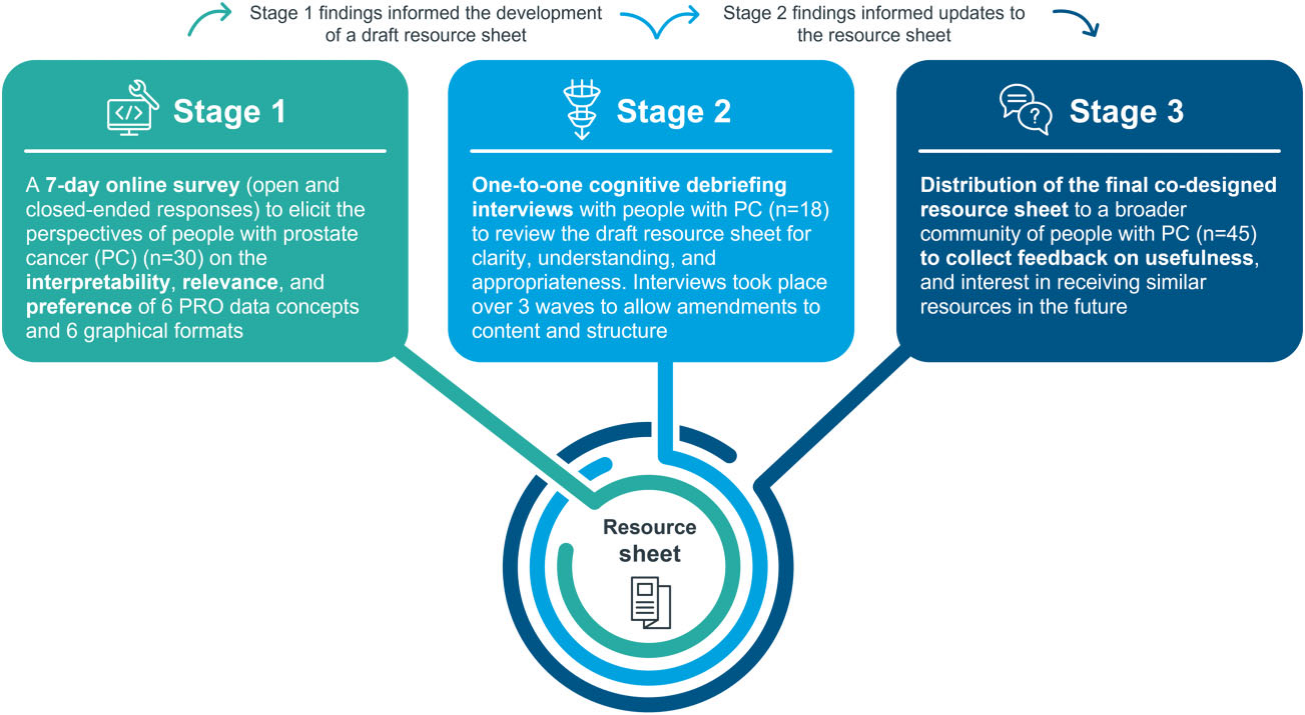
Study design. Stage 1 tested multiple PRO concepts in multiple presentation formats, using an online survey to obtain patients’ assessment of clarity, understandability, and relevance. Survey findings allowed the development of a draft resource sheet to be reviewed and refined during stage 2, where cognitive debriefing interviews took place with additional PC patients. In stage 3, the refined resource sheet was made available to a larger group of PC patients for broader feedback using an open and closed-ended response survey. PC: prostate cancer; PRO: patient-reported outcome.

This three-stage study used a mixed methods cross-sectional design to obtain PC patients’ perspectives of PRO data from the Janssen COU-AA-302 trial dataset, a phase 3, multinational, randomized, double-blind study involving asymptomatic or mildly symptomatic chemotherapy-naive patients with progressive metastatic castration resistant PC (mCRPC) (NCT00887x198).^25^ Access to patient-level data was granted by the Yale University Open Data Access (YODA) Project. The study was approved by WCG IRB (Protocol No. ISOQOL2022). Each stage informed subsequent research stages (Figure 1).

### Patient sample

Participants were invited through emails issued to PCR’s patient community. Stage 1 and 2 participants were screened online, provided written informed consent, and were compensated for their time. In stage 3, the final resource sheet was sent alongside an online survey. It was possible for participants to complete stage 3 even if they had previously been involved in stage 1 or stage 2. No consent was required, or renumeration provided, in stage 3. Full details of the patient sample, including eligibility criteria, are provided in Supplementary Appendix S1.

### Materials and procedures

**Figure 2.**
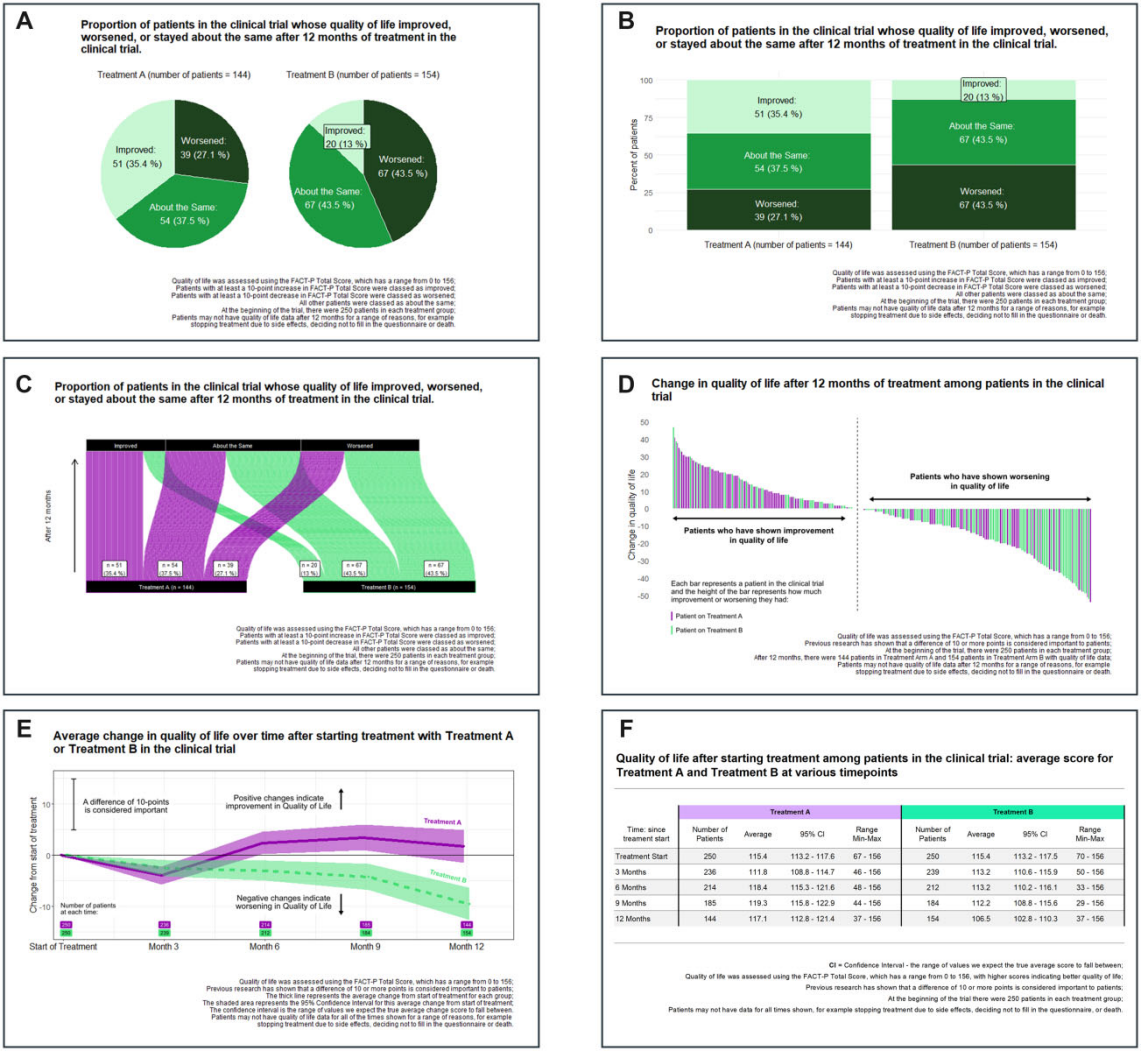
Sample figures presented to patients in stage 1. Example figures displaying the total score changes using a (A) pie chart; (B) stacked bar chart; (C) Sankey diagram; (D) waterfall plot; (E) line graph; (F) data table. Data shown are simulated and not taken from the COU-AA-302 trial. The line graph and data table display group-level change over time (“population-level longitudinal continuous data”), the waterfall plot displays individual level change between baseline and end of treatment (“person-level categorical change data”), and the bar chart, pie chart, and Sankey diagram display the proportion of patients who experienced a meaningful change between baseline and end of treatment (“population-level categorical change data”; meaningful improvement, meaningful worsening, or no meaningful change).^28^

In stage 1, eligible consenting patients participated in a 7-day online survey. The survey assessed perceived relevance of six different PRO data concepts (aligned with COU-AA-302 and using the Functional Assessment of Cancer Therapy-Prostate [FACT-P],^26^ a standard PRO assessment tool implemented in PC clinical trials: social/family well-being, emotional well-being, functional well-being, physical well-being, specific PC concerns, and overall QoL) collected in the COU-AA-302 trial, and the interpretability of six different presentation formats (Figure 2). Figures were rendered in R using the “ggplot” package. Each format was stratified by treatment arm. Footnotes were included to note the meaningful change threshold for the specific scale.

On days 1 through 6, participants received one PRO data concept in one presentation format per day. The PRO data and format differed daily, and the combination was randomized (6 types of data×6 presentation formats = 36 unique combinations). A Latin-square design was used to assign the 36 unique images to participants,^27^ who were divided into six groups. This design was chosen to ensure that each group received each combination of PRO result and presentation format, while controlling for order effects. Given the aims of the study, a full factorial assessment of the conditions among participants was considered unnecessary and would have resulted in additional burden. Participants responded daily (open and close-ended response) to ascertain understanding of the PRO data, clarity of the presentation format, and relevance of the PRO data (Supplementary Appendix S1).

On day 7, participants were presented with one piece of PRO data (randomized) in all six presentation formats and asked to rank them 1 to 6 according to preference. An open-ended response allowed additional comments to be provided. Data were collected through an online survey platform. Responses obtained during days 1 through 7 were used to prepare a draft resource sheet.

In stage 2, participants reviewed a digital version the resource sheet and took part in one-on-one web-based semistructured cognitive debriefing interviews (45–60 min). Interview questions focused on the clarity and interpretation of the text and graphical displays describing PRO data and the usefulness of the resource sheet to facilitate and support treatment discussions with their clinicians. Interviews were conducted in three waves and were audio-recorded and transcribed. After each wave, feedback was analyzed to inform changes to the content and structure of the resource sheet and plan for subsequent interviews.

In stage 3, the finalized resource sheet was circulated via PCR’s Facebook page and newsletter to their patient community mailing list to assess perceptions of usefulness of the information and interest in receiving similar information in future (Supplementary Appendix S1).

### Analysis

Responses from screening were used to describe the study samples. Descriptive statistics were generated for the closed-ended survey questions.

Stage 1 open-ended responses were scored using a 1- to 4-point rating system to assess participants’ understanding of the data (1 = Clear understanding of the data presentation; 2 = Response aligns with the researchers’ intended interpretation in some ways; 3 = Appropriately interpreted the overall message, but response reveals some confusion; 4 = No understanding; N/A = No attempt to understand). Data were independently coded by two researchers (MR and AW [acknowledged]) and aligned with a third researcher (AM) as necessary.

Thematic analysis was used to identify and analyze salient themes that emerged during stage 2 interviews. An experienced qualitative researcher (JR) inductively coded all transcripts in MAXQDA to develop an initial codebook, which was later revised to collate specific segments into general themes. The final codebook was checked for accuracy against the raw data, and themes were analyzed to identify patterns and provide meaningful insights into the perspectives of participants. The coding was reviewed by a senior scientist (FGS) and regular debriefs between moderators (JR, ER, and FGS) provided quality assurance throughout the coding and analysis process. Survey data collected in stage 3 were descriptively analyzed, and participant responses to open-ended survey questions were extracted to support the analysis.

## RESULTS

### Demographics and clinical history

Baseline demographics were broadly comparable across all three stages (Table 1). Stage 1 recruited 30 participants, half of whom had at least some college or university education. Participants reported a range of current PC statuses, with 23.3% being in remission, and 26.7% indicating they were cancer free. Because the majority of participants in stage 1 had a higher education background (70% college or postgrad), greater effort was made to recruit a more representative sample for the following stage. Stage 2 recruited 18 participants, all of whom had received at least a secondary school education up to 16 years; 16.7% were cancer-free at the time of the study, and over 75% had received two or more treatment options. In stage 3, 45 people completed the survey. Respondents reported a range of educational levels, and all stages of PC were represented, with the largest percentage reporting they had early stage, localized PC.

**Table 1.** Patient sample summary

	Stage 1 (n = 30)	Stage 2 (n = 18)	Stage 3 (*n* = 45)
Demographic characteristics			
Age, mean (min–max)			
Years^a^	69.47 (59–83)	66.44 (47–81)	69.07 (45–84)
Gender, *n* (%)			
Male	30 (100%)	18 (100%)	45 (100%)
Trans or nonbinary: assigned male at birth	0	0	0
Ethnicity, *n* (%)			
Asian/Asian British	0	1 (5.6%)	0
Black/African/Caribbean/Black British	1 (3.3%)	1 (5.6%)	0
White	28 (93.3%)	15 (83.3%)	44 (97.8%)
Mixed/multiple ethnic groups	0	1 (5.6%)	0
Other	1 (3.3%)	0	1 (2.2%)
Country, *n* (%)			
England	24 (80.0%)	17 (94.4%)	(not collected)
Scotland	2 (6.7%)	0	
Wales	4 (13.3%)	1 (5.6%)	
Northern Ireland	0	0	
Level of education, *n* (%)			
Primary school	1 (3.3%)	0	0
Secondary school up to 16 years (O-levels, GCSEs, CSE, etc)	3 (10.0%)	7 (38.9%)	6 (13.3%)
Higher or secondary or further education (A-levels, BTEC, etc)	5 (16.7%)	7 (38.9%)	5 (11.1%)
College or university	15 (50.0%)	3 (16.7%)	19 (42.2%)
Postgraduate degree	6 (20.0%)	1 (5.6%)	15 (33.3%)
Employment status, *n* (%)			
Employed by an organization	4 (13.3%)	3 (16.7%)	(not collected)
Self employed	0	4 (22.2%)	
Unemployed, and looking for employment	0	0	
Unemployed, and not looking for work at the moment	2 (6.7%)	0	
Retired	24 (80.0%)	11 (61.1%)	
Student	0	0	
Homemaker	0	0	
Carer of another household member	0	0	
None of these	0	0	
Clinical characteristics			
Current stage of prostate cancer, *n* (%)			
Early stage, contained within the prostate	3 (10.0%)	2 (11.1%)	13 (28.9%)
Locally advanced, has spread just outside the prostate	5 (16.7%)	6 (33.3%)	10 (22.2%)
Advanced metastatic, has spread to the lymph glands or other distant parts of the body	5 (16.7%)	0	10 (22.2%)
Not sure	2 (6.7%)	1 (5.6%)	5 (11.1%)
Cancer free	8 (26.7%)	3 (16.7%)	2 (4.4%)
In remission	7 (23.3%)	6 (33.3%)	5 (11.1%)
Time since original diagnosis, mean (min–max)			
Years	5.54 (1.08–14.92)	4.54 (0.92–19.67)	4.24 (0.08–22.83)
Treatment received, *n* (%)			
Radiotherapy or brachytherapy	14 (46.7%)	12 (66.7%)	19 (42.2%)
Hormone therapy	16 (53.3%)	14 (77.8%)	27 (60.0%)
Surgical removal of the prostate gland	16 (53.3%)	8 (44.4%)	11 (24.4%)
Transurethral resection of the prostate (TURP)	0	1 (5.6%)	6 (13.3%)
High-intensity focused ultrasound (HIFU)	0	0	1 (2.2%)
Cryotherapy	0	0	0
Chemotherapy	3 (10.0%)	0	9 (20.0%)
Steroids	1 (3.3%)	1 (5.6%)	7 (15.6%)
Other^b^	1 (3.3%)	1 (5.6%)	3 (6.7%)

a Stage 1: 97.0% of participants were ≥60 years of age; stage 2: 72.2% of participants were ≥60 years of age; stage 3: 95.6% of participants were ≥60 years of age.

b Stage 1: abiraterone; stage 2: stereotactic ablative radiotherapy (SABR).

### Stage 1—a 7-day online survey

**Figure 3.**
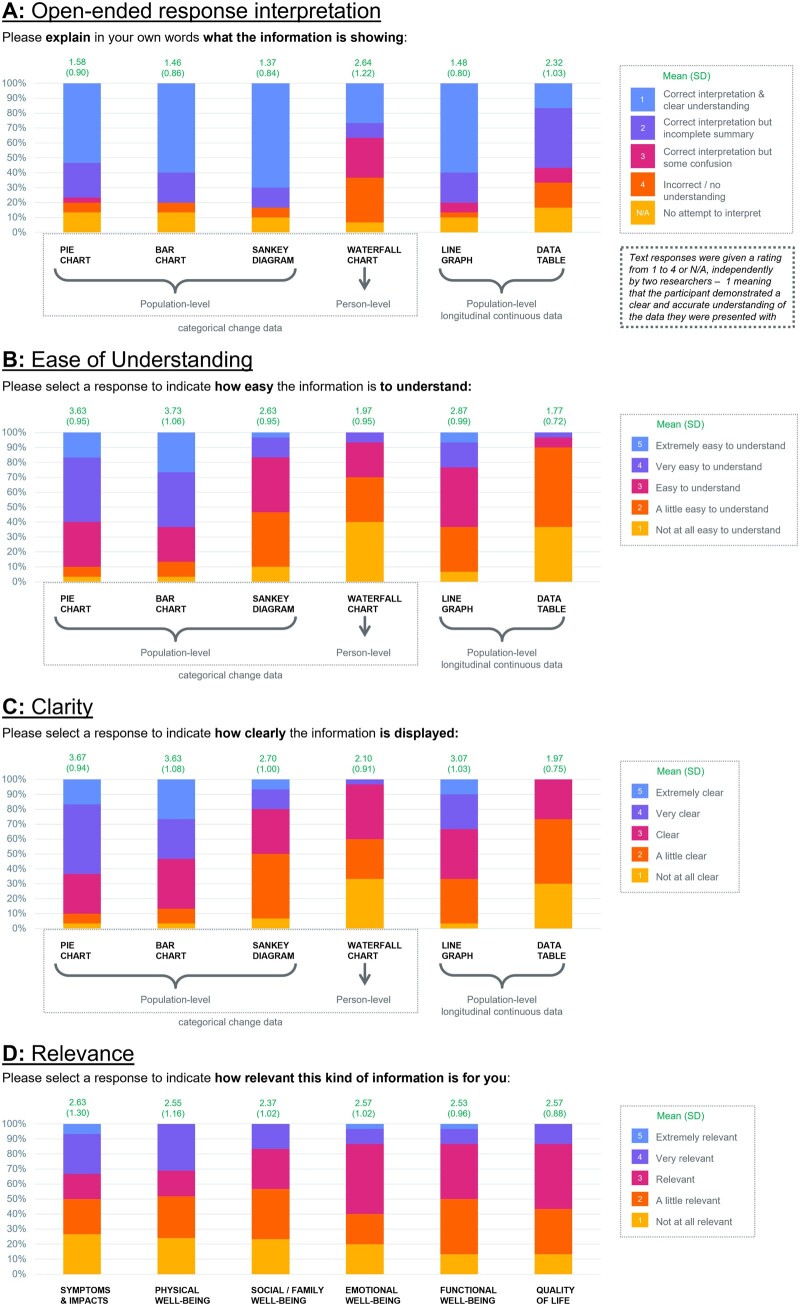
Understandability, clarity, and relevance of PRO data in different presentation modes in stage 1. Figures displaying the distribution of participant responses to stage 1 survey questions. (A) Open-ended interpretation; (B) ease of understanding; (C) clarity; (D) relevance. PRO: patient-reported outcome.

Six data presentation formats (data table, line graph, stacked bar chart, pie chart, Sankey diagram, and waterfall plot) were assessed.^28^ Open-ended responses indicated a high level of understanding of all data presentations except for the waterfall plot and data table. Most accurate interpretation was of the bar chart, pie chart, Sankey diagram, and line graph (Figure 3A).

When asked about understanding and clarity of design, participants considered population-level longitudinal continuous data presentations and population-level categorical change data presentations clear and easy to understand when presented in a line graph, pie chart, and bar chart. Person-level categorical change data presentation using the waterfall plot was seen as less clear and harder to understand (Figure 3B and C).

The six PRO concepts were also assessed for personal relevance. There was no consensus between participants on which concept was most relevant—mean scores for relevance were similar across all six concepts (Figure 3D).

**Figure 4.**
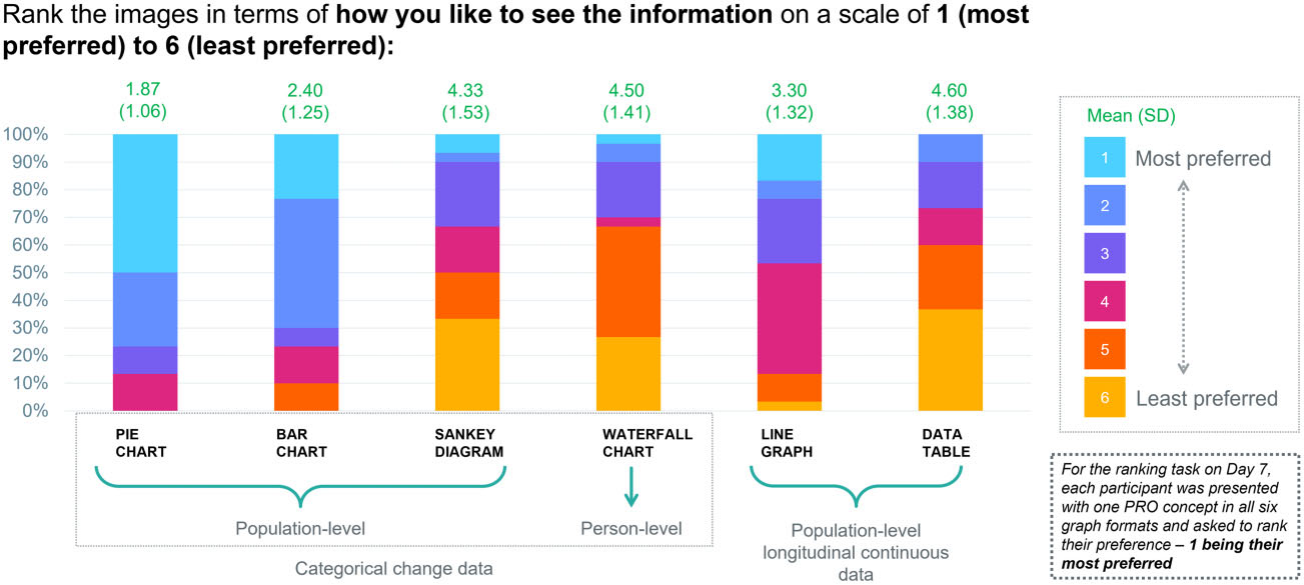
Preference for presentation mode in stage 1. Figure displaying the distribution of participant responses to stage 1 survey question to rank PRO data display formats from 1 (*most preferred*) to 6 (*least preferred*). PRO: patient-reported outcome.

When asked to rank the presentation modes in order of preference, respondents ranked population-level categorical change data presentations in the top three more than population-level longitudinal continuous data or person-level categorical change data presentations (Figure 4); of the population-level categorical change data presentations, pie charts were ranked in the top three more than bar charts (86.7% vs 76.7%). Although participants indicated a preference for the categorical change data in general, several alluded to the relevance of supplementing this with longitudinal data due to the inability of categorical data to show the effect of treatment over multiple time points (Table 2). Of the population-level longitudinal continuous data presentations, line graphs were preferred more often than data tables.

**Table 2.** Preference for cross-sectional data supplemented by longitudinal data

Respondents expressed a preference for cross sectional over longitudinal data
“Image 3 [LINE GRAPH – physical well-being; see Figure 2, Panel E for graph type] gave a breakdown of percentages over the period in months which could also be useful.” Participant 4417
“The image at 4 [LINE GRAPH – emotional well-being; see Figure 2, Panel E for graph type] shows a trend of the treatments and if this was the aim then this would be closer to position 1.” Participant 4420
“[PIE CHART – functional well-being; see Figure 2, Panel A for graph type] does not show the progression of well-being over the 12 months, neither does it show the highs and lows of well-being experienced by some patients.” Participant 4371
“Image 2 [LINE GRAPH – emotional well-being; see Figure 2, Panel E for graph type]—clear comparative beneficial/detrimental of each treatment and covers each of the Quarters and number of patients remaining.” Participant 4403

A preliminary draft resource sheet was constructed containing data representing all six PRO concepts, given that all were deemed relevant in stage 1. Data were presented using a combination of six pie charts (population-level categorical change data)—one for each PRO concept—and one line graph (population-level longitudinal continuous data) for overall QoL. Pie charts were selected because, although pie and bar charts performed similarly in clarity and understanding in stage 1, pie charts were ranked higher in preference. For additional detail about the preliminary resource sheet and subsequent iterations, see Supplementary Appendix S2.

### Stage 2—cognitive debriefing interviews

Feedback was elicited for the graphs, text, and structure of the resource sheet over three interview waves, each containing five to eight participants. Wave 1 (*n* = 5) feedback suggested that participants generally found the information useful and relevant to their PC experience. All PRO concepts were considered relevant, particularly the social, emotional, functional, and QoL impacts.

Some participants suggested the information was overly complex, and highlighted potential “information overload”; others emphasized the importance of using a more personable tone and avoiding—or clearly defining—scientific and medical terms.

The document is very busy … Speaking from my own experience, you know what to do. It’s the KISS, which is “keep it simple, stupid.” … You just want to be able to look at it and go, “Right, that’s that.” (Participant 003)

It’s saying hormone therapy is a treatment, but it doesn’t work for everybody, but now we’ve got this abiraterone acetate. Why not cut it out and just say, ‘This is a new possible hormone therapy, which we believe could be more effective than what we’ve got already?’ (Participant 006)

All participants requested additional detail about aspects of the study during the interviews; for instance, several individuals asked about side effects, which they described as particularly important given that the most bothersome symptoms and impacts they experienced were treatment related. All wave 1 participants expressed some concerns with the pie charts, indicating that the display was overwhelming. Several explicitly suggested bar charts as a better alternative to pie charts. Longer, descriptive titles were preferred over the more concise titles.

You’ve got six squares underneath [pie charts legend], and it took me a while to connect it all together. (Participant 004)

I think sometimes with a bar graph, it’s easier for the eye to follow … You can do a direct comparison between the very high blue one and the very low green one … I basically think, from a graphics point of view, it’s a lot easier to take in a lot more information from a bar graph. (Participant 003)

The overall messaging of the resource sheet was explored. Some participants found the text relating to control patients, and particularly placebo data, to be emotionally distressing.

I’d say it reads like a horror story … You’re saying the people in Group A lived five months longer and the people in Group B didn’t … I find it quite emotionally upsetting … It’s quite soul destroying looking at Group B. (Participant 003)

Following wave 1, text iterations were implemented, including “softening” language around survival and mortality, and adding further context to explain the study design. Pie charts were retained, and bar charts added as an alternative for discussion in wave 2 (Supplementary Appendix S2).

Wave 2 (*n* = 5) feedback indicated that the revisions had improved the resource sheet. Targeted probing suggested specific improvements to aid receptibility. Generally, participants favored bar charts over pie charts. Participants continued to prefer longer, descriptive titles to the more concise titles. Most agreed that, while percentages on the graphs might be easier to understand than raw numbers, it would be ideal to include both (ie, “*n* (%)”).

Looking at those [bar charts], I think they are better than the pie chart…. you’ve got a clearer picture there. (Participant 010)

[I prefer] probably percentages because that way, then, it doesn’t look like Group B is winning over Group A. (Participant 024)

Overall, participants continued to understand the central messages of the PRO data presented but also continued to speak about the importance of side effects in the treatment decision process, requesting more detailed information about them.

If I said I want Treatment A, what are going to be the likely side effects? … For me, that’s been an important issue, knowing what they could be and potentially who to contact and how to mitigate them. (Participant 014)

Further revisions were implemented based on wave 2 feedback (Supplementary Appendix S2). Bar charts were selected as the primary presentation method, with labels showing number and percentage in each block (the original pie charts were shown as an alternative).

In the final wave of interviews (*n* = 8), participants generally thought the content was clear, relevant, and understandable. Most expressed a clear preference for bar charts, with one participant indicating that pie charts seemed too corporate.

I think pie charts are a business thing … ‘These are your sales last year. These are your sales this year.’ … It’s all just very factual … I think it could be delivered a bit softer … You’re talking about people’s journey with cancer here. (Participant 024)

For the longitudinal data, most participants struggled to understand the line graph or did not find it helpful. Although both percentages and raw numbers were found to be acceptable labels, several participants noted that presenting percentages/proportions instead of raw numbers (with “n” in parentheses) would be better, as there were unequal numbers of patients per treatment group. Finally, both older and younger participants spontaneously recommended including information about the ages of the study participants to help contextualize what was perceived as a lackluster survival outcome.

I think this part of it [the line graph] doesn’t actually help because in the first instance, it was quite — it was [already] fairly clear that there was an improvement in quality of life with Group A as distinct from Group B … I think this, to me, actually blurs the information. (Participant 001)

The audience of this information [in the line graph], would they find that important to read? … [It states that] ‘a difference of 10 points is considered important.’ Why is it considered important? To me, as the audience, it’s not important. (Participant 023)

Final revisions were made in line with wave 3 feedback, including minor revisions to language and selection of bar charts and line graphs for population-level categorical change data and population-level longitudinal continuous data, respectively (Supplementary Appendix S2).

### Stage 3—online survey to determine broader feedback

In response to the question, “Did the leaflet contain useful information?”, 41/45 (91.1%) answered “Yes” with a minority answering “Unsure” (*n* = 4/45, 8.9%). No participants answered “No.”

In response to the question, “Would you like to receive similar leaflets in the future?”, 43/45 (95.6%) answered “Yes” with the rest answering “Unsure” (*n* = 2/45, 4.4%). No participants answered “No.” Of those who answered “Yes,” 3/43 (7.0%) said they would like to receive similar leaflets only if they participated in the clinical trial described in the leaflet, whereas the rest (*n* = 40/43, 93.0%) said that they would like to receive similar leaflets even if they had not participated (Table 3). Reasons given (in open-ended responses) related to being interested in recent developments or breakthroughs and new treatments, being interested in clinical trials and scientific research, wanting to understand prognosis, wanting to plan for the future, and wanting to share information in an understandable way with family members.

**Table 3.** Reported reasons people with PC find resource sheets useful

Resource sheet utility
“Useful to simplify results of important trials rather than reading whole paper” Participant 4251
“The more information I have, the better I am able to take decisions about my health and treatment” Participant 4248
“I would like to keep informed about new developments in treatmentcof (sic) prostate cancer” Participant 4230
“It is always good to keep up to date with new treatment progress” Participant 4233
“The more I know the more easily I can get on with my daily life and prepare for the future without fear and anxiety” Participant 4267

Two participants offered additional thoughts on the relevance of the presented data.

Small population studied … Not sure how statistically significant your conclusions are (Participant 4275)

Looking at the QoL data it worries me why the number of responders dropped off massively in group B vs group A! There seems to be a missing story here that without reading the full paper makes me question the validity of the findings (Participant 4303)

One of these participants also indicated a desire for more information in the leaflet pertaining to treatment comparisons and the population studied.

I would have appreciated knowing what was so different about this new medicine vs existing treatments. I would like to have known the name of the study and the ethnicity of those who took part given that prostate cancer possibly has the greatest racial inequality regarding prevalence. (Participant 4303)

Based on this feedback, authors proposed to update the resource sheet to include further information on the population in the clinical trial, but this information was unavailable.

The final resource sheet is available from https://bit.ly/3CS6L0b.

## DISCUSSION

The increasing emphasis on patient involvement in healthcare decision-making has centered around the importance of using patient input to find solutions to the high cost of drug development and societies’ growing healthcare needs.^29^ This involvement has in turn encouraged patients to proactively seek out information about treatment options. However, existing research has emphasized the limitations of healthcare information presented in popular sources, such as social media and online communities.^7–9^

To address patient information needs, this three-stage patient-centered study explored patients’ perspectives on the design of a resource sheet summarizing PC clinical trial data. Overall, participants found the information contained in the resource sheet to be useful, easy to interpret, and relevant to their PC experience. Many were enthusiastic about sharing clinical trial data with their healthcare providers and, in general, this iterative and collaborative approach to codesigning visual clinical trial data lay summaries is likely to benefit patients by supporting patient–clinician discussions about treatment options.

Our findings broadly align with more general evidence from beyond COA disciplines, for instance noting the importance of judicious use of color and other design principles, and observing patient preference for percentiles in communicating quantifiable results.^20^ This study also replicated previous work within the field of PRO research demonstrating that participants’ preferences for certain figure types were not always clearly associated with their ability to understand the data.^17^ Varying levels of scientific knowledge and familiarity with data visualizations between individuals, as well as systemic disparities in health literacy among socioeconomic groups, may have impacted the ability of some to accurately interpret the information presented. Future efforts to develop accessible patient-facing lay summaries should pay close attention to individual and group differences in health literacy.^30^

In this study, different graphs were used to display the same underlying information. However, when using a different type of graph, the information displayed can also change. For example, a pie chart or a bar chart displays proportions at a single time point, whereas a line chart shows average change over time. A comparison of participant preference of these graph types is initially jarring, as they are two very different displays with very different purposes. However, the preference elicited informs as to the *type* of results patients would like to have access to. Regarding the aforementioned example, patients preferred seeing cross-sectional proportions but mentioned that change over time would also be useful context. Therefore, comparing the different graph formats is justified when the aim is to elicit patient preference for the type of data they would like to receive, rather than their ability to interpret the results displayed. Ranking data visualizations must be done in a way that is considerate of the different roles and information that each type provides, while also integrating patient perspectives.

Although participants generally understood the resource sheet and appreciated the objectivity and scientific rigor of the data presented, most contextualized their interpretation based on emotional impact and perceived relevance to their specific experiences. This confirms previous evidence indicating that patients process information and make decisions in ways that are mediated by emotional response.^31^ In our study, even when the information was accurately interpreted, some participants objected to *how* it was presented, questioning the appropriateness of excessively clinical data visualizations when communicating with cancer patients. For example, while participants generally understood the reasons for control groups, the portrayal of untreated patients was nevertheless distressing to some.

Other participants were critical of scientific assumptions about what matters and expressed interest in data that was more relevant to their experience. Some participants suggested that established clinically meaningful changes may not be equally meaningful to all patients; others stated a preference for more detailed information about specific side effects rather than the domain-level PRO data presented. These findings align with previous research on how the differing priorities of patients and researchers can create barriers to meaningful engagement.^32^ Efforts to communicate scientific findings to patient populations should also take *their* priorities and concerns seriously. More research is needed to identify specific strategies for sharing depersonalized data from clinical trials about what is an intensely personal experience for most patients in ways that are both clear and compassionate.

In general, this research has shown that even with the important principles laid out in the developing literature on communicating data to patients,^16^^,^^17^^,^^33^^,^^34^ a flexible, creative, and collaborative approach is essential. This requires a willingness to consult with patients and respond to their feedback in an iterative process, but the resultant resource sheet will more adequately meet patients’ informational and emotional needs.

### Limitations

The results of this study demonstrate an effective method to develop a patient-friendly resource sheet. However, a key finding of this research was that the messaging and communication style of any data summary should remain highly audience specific. Participants in this study came from a variety of educational and socioeconomic backgrounds and had experienced a range of PC disease states; all participants were living in the UK and were predominantly White. Thus, the generalizability of this research is limited by the specific needs and characteristics of those participating. Even within our own study, we observed differences in preference and ability between stages 1 and 2, which may in part be attributable to the greater proportion of people with a higher education background in stage 1 compared to stage 2. Performing the same development exercise, but with a different participant sample, disease area, disease state or treatment journey, may render a resource sheet that differs in the verbal and visual content used to illustrate the clinical trial PRO data. In addition, we appreciate that we tested only six visualizations of PRO data and that these were not systematically selected, but rather informed by prior recommendations, knowledge and experience of the authors, and a review of the literature.^16–19^^,^^33–36^ Other visualizations may also be relevant and helpful to enhance patient understanding, although other research has recently supported the use of line graphs and bar graphs, suggesting that “unfamiliar visuals” (eg, radial bar charts, heat maps, pyramid charts) and additional details on graphs (eg, confidence intervals, norms, missing data) are likely to reduce rather than enhance understanding.^37^

One of the basic principles underpinning the current study was the codevelopment of the resource sheet by social science researchers at IQVIA and researchers and patients at PCR. The social science researchers at IQVIA have a great deal of experience in designing, implementing, analyzing and synthesizing PRO measures, and in presenting data to various audiences. The researchers at PCR have a great deal of knowledge about PC generally, and a good understanding of common requests and complaints among people seeking care for their own or their loved one’s cancer. The patients have firsthand experience of searching for and reading information about treatments, engaging with healthcare professionals, and making decisions about their own treatment and care. The research process enabled all parties to provide valuable information in the design and finalization of the resource sheet. In particular, researchers at IQVIA and PCR were able to work together on all aspects of the study, from protocol development through to result interpretation. Engaging patients was relatively easy, with many willing to participate in all phases of the research when approached through PCR’s patient community. However, reaching underserved and illiterate populations was difficult, and despite broad recruitment channels (including online and offline approaches), the sample was still relatively educated and may not necessarily be considered broadly representative of the UK’s PC population. In consideration of this, while the current study developed a simple digital resource sheet, a paper leaflet may be considered in future to be more inclusive for aging or disadvantaged populations. Conversely, a fully digital tool might allow for more tailored, individualized messaging.

### Future directions

The findings outlined above suggest several areas that would benefit from further research. Degree of detail and scope of content should be further evaluated (eg, specific symptoms and impacts rather than grouping in categories or domains), as well as reporting by subgroup (eg, age, race, ethnicity), to understand the experiences of diverse groups of patients.

Participants also emphasized the importance of information that was humane and empathetic and that might be used to make healthcare decisions. Additionally, it is essential that this information is perceived as credible by patients, and thus should be presented in an appropriate forum where perceptions of bias are minimized.^38^ For example, on a website hosted by regulators, professional organizations, healthcare systems, or patient advocacy groups, rather than insurance or biopharmaceutical companies. In future, the inclusion of qualitative data might increase the accessibility of quantitative data and reduce the likelihood that patients will feel alienated from, or objectified by, such data.

Further, it will be important to explore avenues of direct to patient communication for PRO data that are both compliant with regulations, reach broad audiences, are relevant, and are perceived as valuable to both healthcare professionals and patients. Unfortunately, not all healthcare professionals currently see value in PRO data for clinical care. A recent survey among oncologists showed a generally favorable, but not enthusiastic attitude to PRO data, although it also showed that those more familiar with PRO data generally had more positive attitudes.^39^ Other research has shown that healthcare professionals who have read research outlining the impact of the treatment on patients’ overall QoL will likely approach consultations with more empathy,^40^ will ask patients about their treatment views and preferences,^41–43^ and will use these data to set and manage expectations with patients should that treatment be selected for use.^44^

Although every effort should be made to meet patients where they are, future work might also reference prior efforts to investigate the use of tutorials to increase visualization literacy in lay audiences.^45^^,^^46^ The development of standardized assessment tests of visualization literacy would help prepare materials that consider variations in the complexity of visual designs and how they can be adapted to meet the needs of patients with varied literacy levels.^13^ A variety of methods of dissemination, beyond static resources like our developed resource sheet, should be considered in the future, including exploring the use of web-based platforms or “dashboards,” allowing patients to find answers to questions by filtering data according to a range of criteria (eg, symptoms/impacts, demographic characteristics).

## CONCLUSION

Taken together, our findings advance the understanding of the types of data patients are interested in, visualizations that are preferred by and promote patient understanding, and dissemination preferences. Critics of the “medical gaze,” which has sometimes resulted in clinicians failing to hear and attend to the individual needs reported by patients, have emphasized the importance of more patient-centric models in healthcare delivery and clinical research.^47^^,^^48^ This study confirms the urgent need, in a landscape where patients are increasingly recognized as coequal stakeholders, for researchers, regulatory authorities, and drug manufacturers to take seriously the voices and concerns of patients, who ask questions and demand answers that are as sensitive and empathetic as they are clear and relevant.

## Supplementary Material

ocad099_Supplementary_Data

## Data Availability

The data underlying this study are available from the authors, upon request. Some restrictions apply to the availability of patient-level data provided by the Yale University Open Data Access (YODA) Project, which were used under license for this study.
