# Supplementary material for: A powerful partnership: researchers and patients working together to develop a patient-facing summary of clinical trial outcome data
Source: J Am Med Inform Assoc. 2023 Jun 21;31(2):363–74. doi: 10.1093/jamia/ocad099 (PMC10797263; doi:10.1093/jamia/ocad099)
Supplement: ocad099_Supplementary_Data [file ocad099_supplementary_data.zip › ocad099_Supplementary_Data/SuppA_Supplemental Methodology_final_27Apr.docx]

**Supplemental Methodology**

**Patient Recruitment Criteria**

In Stages 1 and 2, patients were invited to participate through e-mails issued by PCR to their members. Patients interested in participating were directed to a website where they completed a screening and demographic form to determine eligibility. Patients who qualified to participate in the study based on their screener responses were directed to a website containing detailed study information and a consent form. The target was to recruit 30 participants for Stage 1 and 15-20 participants for Stage 2. Participants were able to take part in one or several stages of the study.

Stage 1 respondents were screened online based on the following criteria:

- born male,
- residing in the UK (England, Scotland, Wales, Northern Ireland),
- aged ≥18 years,
- diagnosed with PC,
- fluent in English,
- with confirmed access to a computer, internet-enabled phone, or tablet.

In Stage 2, respondents were eligible if they met all Stage 1 criteria and agreed to be audio-recorded during cognitive debriefing interview.

Patients were excluded from Stage 1 and Stage 2 if they self-disclosed cognitive deficits or visual impairments precluding them from participating in the study.

All Stage 1 and 2 participants provided written informed consent and were compensated for their time.

In Stage 3, the resource sheet was sent to members of PCR’s patient community (approximately 6500 individuals) via newsletter and posted on PCR’s Facebook page, with an invitation to provide feedback though an online survey. No consent was required, and no remuneration was provided to Stage 3 participants.

**Stage 1 Survey Probes and Response Options**

***Days 1-6***

*Understanding:*

1) Explain the display in your own words (open-ended response);

2) State which of two treatments is performing better (“A”, “B”, “Not sure”);

3) Describe the ease of understanding of the information presented (“Not at all easy to understand”, “A little easy to understand”, “Easy to understand”, “Very easy to understand”, “Extremely easy to understand”);

*Clarity of format:*

4) Describe the clarity of the information displayed (“Not at all clear, “A little clear, “Clear, “Very clear, “Extremely clear”),

*Relevance of PRO data:*

5) Describe the personal relevance of the information displayed (“Not at all relevant”, “A little relevant”, “Very relevant”, “Extremely relevant”).

***Day 7***

*Preference:*

1) Rank the images (*images provided*) in terms of how you like to see the information (1 [your most preferred way of seeing the information] through 6 [your least preferred way of seeing the information]).

2) Explain why you prefer the image you rated as number one over the other images (open-ended response).

**Stage 3 Survey Probes and Response Options**

1) Did the leaflet contain useful information?

a. Yes

b. No

c. Unsure

2) Would you like to receive similar leaflets in the future?

a. Yes – but only if I have participated in the clinical trial described in the leaflet

b. Yes – even if I have not participated in the clinical trial described in the leaflet

c. No

d. Unsure

3) Why or why not? (open-ended response).
