# Supplementary material for: A powerful partnership: researchers and patients working together to develop a patient-facing summary of clinical trial outcome data
Source: J Am Med Inform Assoc. 2023 Jun 21;31(2):363–74. doi: 10.1093/jamia/ocad099 (PMC10797263; doi:10.1093/jamia/ocad099)
Supplement: ocad099_Supplementary_Data [file ocad099_supplementary_data.zip › ocad099_Supplementary_Data/SuppB_Supplemental Results_final_27Apr.docx]

**Development of the initial resource sheet and subsequent iterative versions:**

The initial draft and subsequent iterations of the resource sheet were developed in collaboration with a professional medical and patient communications and design studio team at IQVIA.

The research team worked with medical writers to develop the written component (main bodies of text) based on the Janssen clinical trial materials and simulated data. Writers had substantial experience creating patient-facing content, including plain language summaries, to ensure content was accessible, inclusive and aligned to core health literacy principles. Considerations of aspects such as reading age, removal of jargon, removing language that could be insensitive, labelling, or dehumanizing, using phonetics to empower patient-clinician discussions with confidence, and the use of short sentences and paragraphs to structure digestible information were all made. Written copy was passed to the design team for layout and graphic design, and graphical data depictions were added based on findings from Stage 1 research, as well as appropriate supportive visual elements such as photos, icons, typography, and colour. Designers and writers worked together closely to ensure designs held health and numerical literacy level principles, followed accessibility guidance, and were focused on patient-centricity – including the use of best practice in font size, colour contract, use of white space, creating structured sections, and using clear, relevant, icons. The original graphs that had been reviewed by participants in Stage 1 (main manuscript, Figure 1) were used as the basis for the initial resource sheet and were redrawn by the design team to ensure all visual elements of the resource sheet were cohesive, of sufficient resolution, and met professional standards for communication design, while also maintaining the scientific integrity of the research.

Following each wave of patient interviews, the research team consolidated patient feedback and provided recommended changes to the resource sheet. These were then shared with the writing and design team via markups, in-file comments, and verbal discussion. The design team implemented changes and advised the researchers on how to meet design industry standards that aligned with patient feedback. Following implementation of patient feedback, the updated resource sheet was tested in subsequent interview waves. Supplemental Table 1 provides additional information on the iterative development process.

*Supplemental Table 1: Development of the final resource sheet was conducted via iteration through interview waves 1, 2, and 3; starting from the initial sheet developed at the end of Stage 1, through Stage 2, to the final sheet that was shared in the Stage 3 survey.*

|  | **What was tested 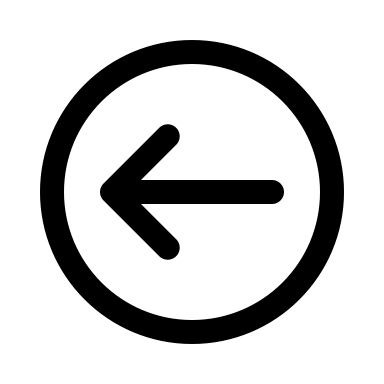** | **Participant feedback 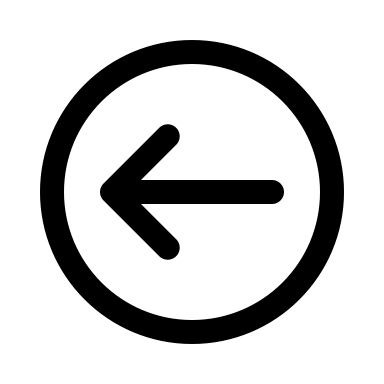** | **Changes made following  review of feedback 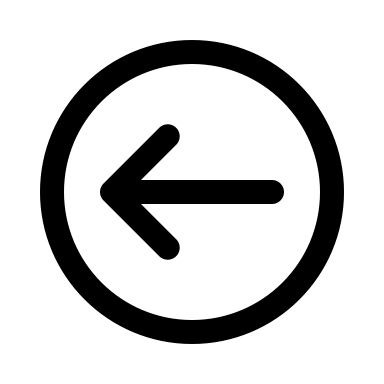** |
| --- | --- | --- | --- |
| **Initial sheet – Stage 1 / Wave 1 – Stage 2** | The initial resource sheet using simulated data developed in Stage 1 was tested with patients in Stage 2-Wave 1 interviews, and contained:   - 6 PRO concepts in two styles of pie charts:  1. Simple pies with simple domain titles i.e.,   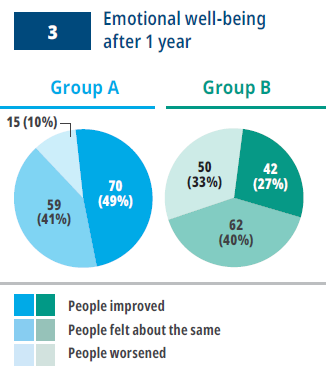   1. Pies with accentuated slices, emphasizing proportions highlighted in descriptive graph titles i.e.,   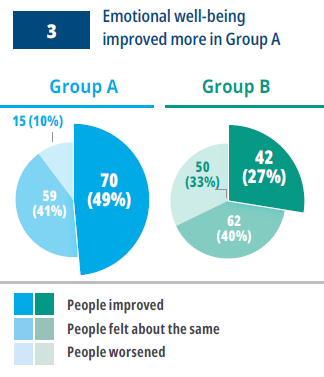   - 1 PRO concept in a line graph with simple domain title   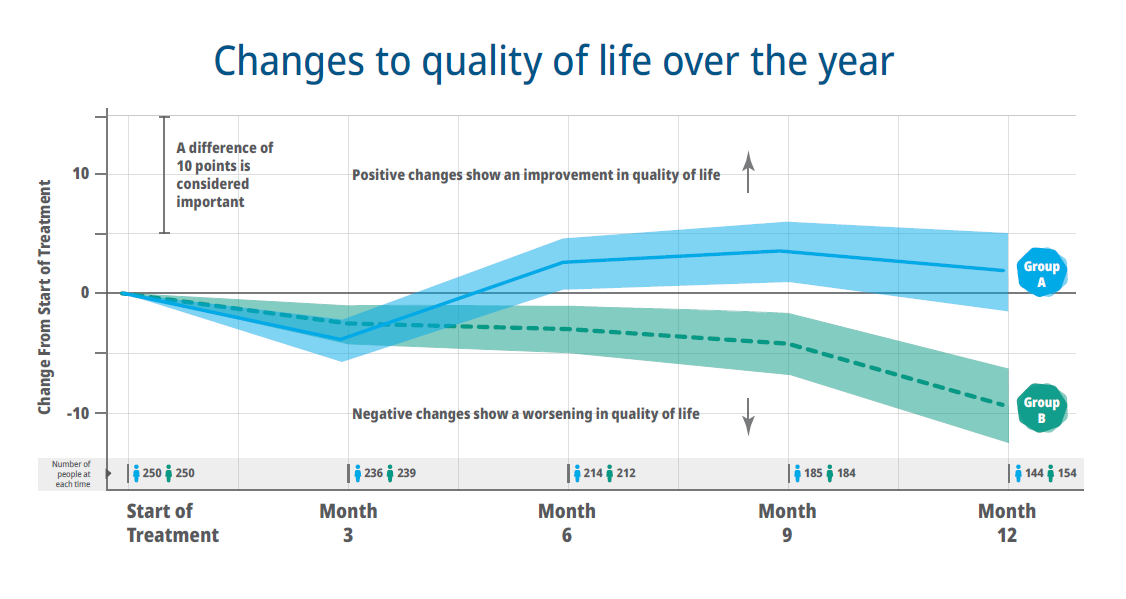 | - All participants expressed concerns with the pie charts (both styles, as above) - Several participants explicitly suggested bar graphs as a better alternative to pie charts - All PRO concepts were considered relevant - Overall, participants found the information useful and relevant to their experience with prostate cancer - Feedback was given on the text, which suggested information was overly complex and contained some unnecessary detail. However, some participants requested additional detail about side effects, which they described as particularly important given that their most bothersome symptoms and impacts were treatment-related | - Removed pie chart style 2 (accentuated) - Added bar charts - Various textual updates to the written components surrounding the graphs |
| **Wave 2 – Stage 2** | Based on Wave 1 feedback, the resource sheet tested in Wave 2 contained:   - 6 PRO concepts in two graph type options:  1. Simple pies with simple domain titles 2. Simple bar charts with simple domain titles i.e.,   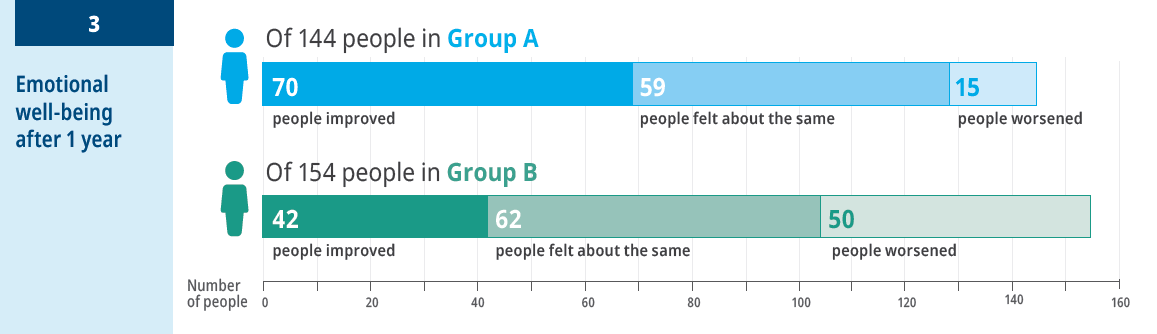   - 1 PRO concept in a line graph with simple domain title (retained) | - Participants preferred the bar charts over the pie charts - Participants wanted longer, descriptive graph titles - Though percentages on the graphs might be easier to understand than raw numbers, it is ideal to include both (i.e., “n(%)”) - Participants continued to speak about side effects as a large part of the treatment decision process and that this could be emphasized further - Overall, participants seemed to understand the central messages of the PRO data | - Descriptive graph titles and percentages were added to the bar charts - The order of display for the bar and pie chart alternatives was changed to show bar charts before pie charts |
| **Wave 3 – Stage 2** | Based on Wave 2 feedback, the resource sheet tested in Wave 3 contained:   - 6 PRO concepts in two graph type options:  1. Bar charts with descriptive graph titles and percentages   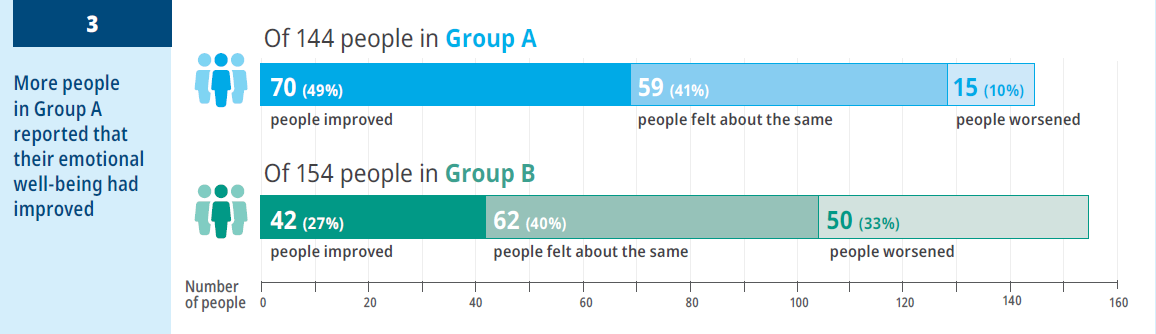   1. Simple pies with simple domain titles  - 1 PRO concept in a line graph with simple domain title (retained) | - Participants generally thought the content was clear, relevant, and understandable - Most participants expressed a clear preference for bar charts, but some noted that presenting percentages/proportions instead of Ns (with Ns in parentheses) would better - Most struggled to understand the line graph or did not find it helpful/necessary - Both older and younger participants spontaneously recommended including information about the ages of the study participants to help contextualize what was perceived as “lacklustre” survival outcomes | - Removed pie charts - Replaced simulated data with real data from the Janssen clinical trial |
| **Final sheet – Stage 3** | Based on Wave 3 feedback, the resource sheet shared with Stage 3 survey contained:   - 6 PRO concepts in bar charts with descriptive titles and percentages   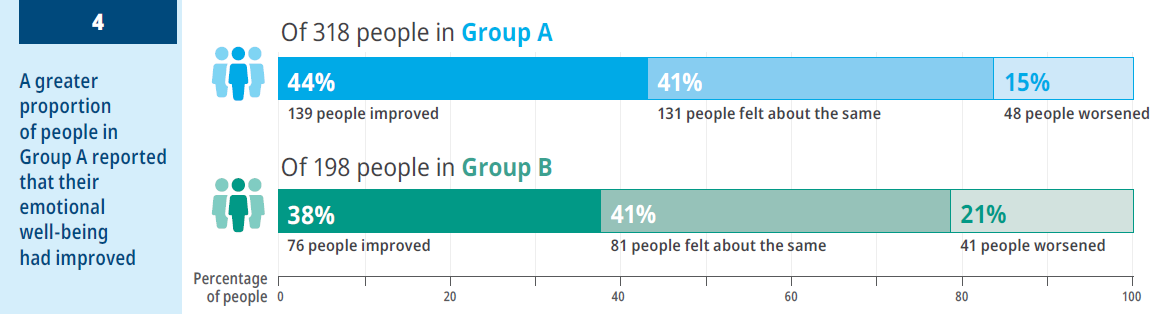   - 1 PRO concept in a line graph with simple domain title (retained) | Stage 3 sought feedback on the overall usefulness and relevance of the whole resource sheet. Feedback on the graphs was not specifically sought in  Stage 3. | No changes following Stage 3 have been made at this time. |
